# Supplementary material for: Selecting Thresholds of Heat-Warning Systems with Substantial Enhancement of Essential Population Health Outcomes for Facilitating Implementation
Source: Int J Environ Res Public Health. 2021 Sep 9;18(18):9506. doi: 10.3390/ijerph18189506 (PMC8471601; doi:10.3390/ijerph18189506)
Supplement: Supplementary file 1 [file ijerph-18-09506-s001.zip › ijerph-1316423-supplementary.pdf]

Table S1. Relative risks (RRs) of (a) heat-related emergency visits, (b) heat-related hospital visits, and (c) all-cause mortality at different WBGT threshold candidates for different sex and age groups.

	RR (95%CI[a])					RaRR
**Threshold (°C)**	**30**	**31**	**31.5**	**32**	**32.5**	
		(a)	***Heat-related emergency visits***			
			Whole Taiwan			
Lag0	1.27***(1.26,1.29)	1.34***(1.32,1.36)	1.38***(1.35,1.42)	1.49***(1.44,1.54)	**1.83***(1.68,1.99)**	**1.44**
Lag1	1.08***(1.07,1.10)	1.10***(1.08,1.13)	1.13***(1.10,1.16)	1.17***(1.13,1.22)	**1.25***(1.14,1.37)**	**1.16**
Lag2	1.00 (0.99,1.01)	1.02* (1.00,1.04)	1.04***(1.02,1.07)	1.07***(1.03,1.11)	1.09 (0.99,1.20)	
			Female			
Lag0	1.22***(1.19,1.25)	1.27***(1.23,1.32)	1.32***(1.26,1.39)	1.45***(1.35,1.55)	**2.06***(1.74,2.45)**	**1.69**
Lag1	1.08***(1.05,1.11)	1.12***(1.07,1.16)	1.15***(1.09,1.21)	1.21***(1.12,1.31)	**1.31** (1.09,1.59)**	**1.21**
Lag2	1.08***(1.06,1.11)	1.12***(1.09,1.16)	1.19***(1.13,1.24)	1.25***(1.16,1.35)	**1.42***(1.19,1.70)**	**1.31**
			Male			
Lag0	1.29***(1.27,1.31)	1.36***(1.33,1.39)	1.41***(1.37,1.44)	1.51***(1.45,1.58)	**1.75***(1.58,1.93)**	**1.36**
Lag1	1.09***(1.07,1.11)	1.10***(1.08,1.13)	1.13***(1.10,1.16)	1.16***(1.11,1.22)	**1.23***(1.10,1.38)**	**1.13**
Lag2	0.98***(0.97,0.99)	0.99 (0.97,1.01)	1.00 (0.98,1.03)	1.01 (0.97,1.06)	1.00 (0.90,1.12)	
			Age 0-14			
Lag0	1.11***(1.06,1.16)	1.17***(1.08,1.25)	1.23***(1.11,1.36)	1.42***(1.20,1.68)	**1.84** (1.20,2.80)**	**1.66**
Lag1	1.07* (1.01,1.13)	1.14** (1.05,1.24)	1.25***(1.12,1.40)	1.40***(1.17,1.67)	**1.94** (1.25,3.00)**	**1.81**
Lag2	1.02 (0.97,1.07)	1.03 (0.96,1.11)	1.04 (0.94,1.15)	1.05 (0.88,1.24)	1.36 (0.87,2.11)	
			Age 15-64			
Lag0	1.28***(1.27,1.30)	1.35***(1.32,1.38)	1.40***(1.36,1.43)	1.50***(1.44,1.56)	**1.81***(1.64,1.99)**	**1.41**
Lag1	1.09***(1.07,1.11)	1.11***(1.08,1.13)	1.13***(1.10,1.16)	1.16***(1.11,1.21)	**1.20***(1.08,1.34)**	**1.10**
Lag2	0.99 (0.98,1.00)	1.00 (0.98,1.02)	1.02 (1.00,1.05)	1.04 (1.00,1.08)	1.03 (0.93,1.14)	
			Age ≥65			
Lag0	1.30***(1.26,1.34)	1.35***(1.29,1.40)	1.38***(1.31,1.46)	1.49***(1.37,1.62)	**1.92***(1.57,2.36)**	**1.48**
Lag1	1.06***(1.03,1.10)	1.09***(1.04,1.14)	1.12***(1.05,1.19)	1.18***(1.08,1.30)	**1.33* (1.07,1.67)**	**1.25**
Lag2	1.07***(1.04,1.10)	1.11***(1.07,1.16)	1.17***(1.10,1.23)	1.23***(1.12,1.34)	**1.35** (1.09,1.67)**	**1.26**
		(b)	***Heat-related hospital visits***			
			Whole Taiwan			
Lag0	1.08***(1.08,1.09)	1.09***(1.09,1.10)	1.11***(1.10,1.11)	1.15***(1.14,1.16)	**1.29***(1.27,1.31)**	**1.19**
Lag1	1.04***(1.04,1.05)	1.07***(1.07,1.07)	1.09***(1.09,1.10)	1.12***(1.12,1.13)	**1.21***(1.19,1.23)**	**1.16**
Lag2	1.05***(1.05,1.05)	1.07***(1.06,1.07)	1.08***(1.08,1.09)	1.11***(1.11,1.12)	**1.17***(1.15,1.19)**	**1.11**
			Female			
Lag0	1.08***(1.07,1.08)	1.08***(1.08,1.09)	1.10***(1.09,1.10)	1.14***(1.13,1.15)	**1.27***(1.24,1.29)**	**1.18**
Lag1	1.04***(1.04,1.04)	1.07***(1.06,1.07)	1.09***(1.08,1.09)	1.12***(1.11,1.13)	**1.20***(1.18,1.23)**	**1.15**
Lag2	1.06***(1.05,1.06)	1.07***(1.07,1.08)	1.09***(1.08,1.09)	1.12***(1.11,1.13)	**1.18***(1.16,1.21)**	**1.11**
			Male			
Lag0	1.09***(1.09,1.10)	1.11***(1.10,1.11)	1.12***(1.12,1.13)	1.17***(1.16,1.18)	**1.33***(1.29,1.36)**	**1.22**
Lag1	1.05***(1.04,1.05)	1.07***(1.07,1.08)	1.10***(1.09,1.10)	1.13***(1.11,1.14)	**1.21***(1.18,1.24)**	**1.15**
Lag2	1.05***(1.04,1.05)	1.06***(1.06,1.07)	1.08***(1.07,1.08)	1.11***(1.10,1.12)	**1.15***(1.12,1.18)**	**1.10**
			Age 0-14			
Lag0	1.08***(1.07,1.09)	1.09***(1.08,1.11)	1.11***(1.09,1.14)	1.15***(1.11,1.19)	**1.24***(1.14,1.35)**	**1.15**
Lag1	1.05***(1.04,1.06)	1.08***(1.06,1.10)	1.11***(1.08,1.14)	1.15***(1.10,1.19)	**1.31***(1.20,1.43)**	**1.25**
Lag2	1.06***(1.05,1.07)	1.08***(1.06,1.10)	1.10***(1.07,1.12)	1.13***(1.09,1.17)	**1.17***(1.07,1.28)**	**1.10**
			Age 15-64			
Lag0	1.09***(1.09,1.09)	1.10***(1.09,1.10)	1.11***(1.11,1.12)	1.16***(1.15,1.17)	**1.30***(1.28,1.32)**	**1.19**
Lag1	1.04***(1.04,1.05)	1.07***(1.06,1.07)	1.09***(1.09,1.10)	1.12***(1.11,1.13)	**1.20***(1.17,1.22)**	**1.15**
Lag2	1.05***(1.05,1.05)	1.07***(1.06,1.07)	1.08***(1.08,1.09)	1.11***(1.11,1.12)	**1.17***(1.15,1.19)**	**1.15**
			Age ≥65			
Lag0	1.06***(1.05,1.07)	1.06***(1.05,1.07)	1.06***(1.05,1.08)	1.10***(1.07,1.12)	**1.23***(1.17,1.29)**	**1.16**
Lag1	1.04***(1.03,1.05)	1.07***(1.06,1.08)	1.09***(1.08,1.11)	1.13***(1.11,1.15)	**1.26***(1.19,1.32)**	**1.21**
Lag2	1.05***(1.04,1.06)	1.06***(1.05,1.07)	1.08***(1.06,1.09)	1.10***(1.08,1.13)	**1.14***(1.08,1.20)**	**1.09**
		(c)	***All-cause mortality***			
			Whole Taiwan			
Lag0	1.00***(1.00,1.01)	1.00* (1.00,1.01)	1.01* (1.00,1.01)	1.01** (1.00,1.02)	**1.03** (1.01,1.05)**	**1.03**
Lag1	1.00** (1.00,1.01)	1.01***(1.00,1.01)	1.01***(1.01,1.02)	1.03***(1.02,1.04)	**1.05***(1.03,1.08)**	**1.05**
Lag2	1.00 (1.00,1.00)	1.00* (1.00,1.01)	1.01* (1.00,1.01)	1.01 (1.00,1.02)	**1.03* (1.00,1.05)**	**1.03**
			Female			
Lag0	1.00 (1.00,1.00)	1.00 (0.99,1.01)	1.00 (0.99,1.01)	1.01 (0.99,1.02)	1.01 (0.98,1.05)	
Lag1	1.01** (1.00,1.01)	1.01***(1.01,1.02)	1.02***(1.01,1.03)	1.03***(1.01,1.04)	**1.05* (1.01,1.09)**	**1.04**
Lag2	1.00 (1.00,1.01)	1.00 (1.00,1.01)	1.00 (1.00,1.01)	1.01 (0.99,1.02)	**1.04* (1.00,1.07)**	**1.04**
			Male			

Lag0	1.01***(1.00,1.01)	1.01***(1.00,1.01)	1.01** (1.00,1.02)	1.02** (1.01,1.03)	**1.05** (1.02,1.08)**	**1.04**
Lag1	1.00 (1.00,1.01)	1.01* (1.00,1.01)	1.01** (1.00,1.02)	1.02***(1.01,1.04)	**1.05***(1.02,1.09)**	**1.05**
Lag2	1.00 (1.00,1.01)	1.01* (1.00,1.01)	1.01* (1.00,1.01)	1.01 (1.00,1.02)	1.02 (0.99,1.05)	
			Age 0-14			
Lag0	1.00 (0.97,1.02)	0.99 (0.95,1.03)	0.98 (0.92,1.04)	1.02 (0.92,1.13)	1.14 (0.88,1.48)	
Lag1	1.00 (0.97,1.03)	1.01 (0.97,1.06)	1.02 (0.96,1.10)	0.97 (0.87,1.09)	0.81 (0.60,1.08)	
Lag2	1.00 (0.98,1.03)	0.98 (0.94,1.02)	0.97 (0.91,1.03)	0.95 (0.86,1.06)	1.12 (0.86,1.46)	
			Age 15-64			
Lag0	1.00 (1.00,1.01)	1.01 (1.00,1.01)	1.00 (0.99,1.01)	1.00 (0.99,1.02)	1.02 (0.98,1.07)	
Lag1	1.00 (0.99,1.00)	1.00 (0.99,1.01)	1.01 (1.00,1.02)	1.02 (1.00,1.04)	1.03 (0.98,1.08)	
Lag2	1.00 (1.00,1.01)	1.01 (1.00,1.01)	1.01 (1.00,1.02)	1.01 (0.99,1.03)	**1.05* (1.00,1.09)**	**1.05**
			Age ≥65			
Lag0	1.00** (1.00,1.01)	1.00* (1.00,1.01)	1.01* (1.00,1.01)	1.02** (1.01,1.03)	**1.03* (1.01,1.06)**	**1.03**
Lag1	1.01***(1.00,1.01)	1.01***(1.01,1.02)	1.02***(1.01,1.02)	1.03***(1.02,1.04)	**1.06***(1.03,1.09)**	**1.05**
Lag2	1.00 (1.00,1.00)	1.00 (1.00,1.01)	1.01* (1.00,1.01)	1.01 (1.00,1.02)	1.02 (0.99,1.05)	

*$p < 0.05$** $p < 0.01$*** $p < 0.001$, [a]CI: confidence interval. RaRR is reference-adjusted risk ratio, defined as RR (with statistical significance) of the threshold candidates against that of a reference (30°C); RaRR is shown only for the highest threshold candidate (in bold).

Table S2. Relative risks (RRs) of (a) heat-related emergency visits, (b) heat-related hospital visits, and (c) all-cause mortality at different WBGT threshold candidates for different sub-regions.

	RR (95%CI[a])						RaRR
**Threshold (°C)**	**30**	**32**	**32.5**	**33**	**33.5**	**34**	
**(a)** ***Heat-related emergency visits***							
Whole Taiwan							
Lag0	1.27***(1.26,1.29)	1.49***(1.44,1.54)	**1.83***(1.68,1.99)**				**1.44**
Lag1	1.08***(1.07,1.10)	1.17***(1.13,1.22)	**1.25***(1.14,1.37)**				**1.16**
Lag2	1.00 (0.99,1.01)	1.07***(1.03,1.11)	1.09 (0.99,1.20)				
North Taiwan							
Lag0	1.18***(1.16,1.19)	1.17***(1.14,1.20)	1.18***(1.14,1.21)	1.22***(1.16,1.28)	1.38***(1.26,1.52)	**2.57***(1.88,3.53)**	**2.18**
Lag1	1.07***(1.06,1.09)	1.09***(1.07,1.12)	1.10***(1.06,1.13)	1.14***(1.08,1.20)	1.24***(1.12,1.38)	**1.88** (1.29,2.74)**	**1.76**
Lag2	1.03***(1.02,1.04)	1.06***(1.04,1.09)	1.07***(1.04,1.11)	1.11***(1.05,1.16)	1.25***(1.12,1.39)	**1.56* (1.03,2.36)**	**1.51**
Central Taiwan							
Lag0	1.25***(1.22,1.28)	1.44***(1.32,1.57)	**2.16***(1.65,2.82)**				**1.73**
Lag1	1.08***(1.05,1.10)	1.08 (0.98,1.18)	1.19 (0.88,1.61)				
Lag2	1.04***(1.02,1.07)	1.08 (0.98,1.19)	1.10 (0.80,1.49)				
South Taiwan							
Lag0	1.35***(1.32,1.38)	1.63***(1.53,1.74)	**2.14***(1.82,2.53)**				**1.59**
Lag1	1.10***(1.07,1.12)	1.23***(1.15,1.33)	**1.65***(1.39,1.97)**				**1.50**
Lag2	0.97** (0.95,0.99)	1.02 (0.95,1.10)	1.09 (0.90,1.31)				
East Taiwan							
Lag0	1.29***(1.25,1.33)	**1.40***(1.28,1.53)**	1.32***(1.13,1.54)	0.90 (0.56,1.44)			**1.09**
Lag1	1.04* (1.00,1.08)	1.03 (0.93,1.14)	1.02 (0.85,1.21)	1.51 (0.96,2.37)			
Lag2	0.98 (0.95,1.01)	1.03 (0.94,1.13)	1.08 (0.92,1.27)	0.78 (0.48,1.29)			
**(b)** ***Heat-related hospital visits***							
Whole Taiwan							
Lag0	1.08***(1.08,1.09)	1.15***(1.14,1.16)	**1.29***(1.27,1.31)**				**1.19**
Lag1	1.04***(1.04,1.05)	1.12***(1.12,1.13)	**1.21***(1.19,1.23)**				**1.16**
Lag2	1.05***(1.05,1.05)	1.11***(1.11,1.12)	**1.17***(1.15,1.19)**				**1.11**
North Taiwan							
Lag0	1.06***(1.06,1.06)	1.05***(1.05,1.06)	1.05***(1.05,1.06)	1.06***(1.05,1.07)	1.09***(1.07,1.11)	**1.30***(1.19,1.41)**	**1.23**
Lag1	1.04***(1.03,1.04)	1.05***(1.04,1.05)	1.05***(1.04,1.05)	1.06***(1.05,1.07)	1.11***(1.09,1.13)	**1.37***(1.28,1.45)**	**1.32**
Lag2	1.05***(1.04,1.05)	1.05***(1.05,1.06)	1.06***(1.05,1.06)	1.07***(1.06,1.08)	1.12***(1.10,1.14)	**1.25***(1.17,1.34)**	**1.19**
Central Taiwan							
Lag0	1.08***(1.08,1.08)	1.13***(1.11,1.14)	**1.31***(1.26,1.36)**				**1.21**
Lag1	1.05***(1.04,1.05)	1.08***(1.07,1.10)	**1.24***(1.19,1.29)**				**1.18**
Lag2	1.05***(1.04,1.05)	1.06***(1.04,1.07)	**1.11***(1.07,1.16)**				**1.06**
South Taiwan							
Lag0	1.12***(1.12,1.13)	1.26***(1.23,1.29)	**1.39***(1.30,1.48)**				**1.24**
Lag1	1.06***(1.05,1.07)	1.16***(1.13,1.19)	**1.30***(1.22,1.39)**				**1.23**
Lag2	1.04***(1.04,1.05)	1.11***(1.08,1.14)	**1.11** (1.04,1.18)**				**1.07**
East Taiwan							
Lag0	1.14***(1.13,1.15)	**1.19***(1.16,1.23)**	1.19***(1.12,1.25)	1.05 (0.89,1.24)			**1.04**
Lag1	1.05***(1.04,1.07)	1.06** (1.02,1.09)	1.06 (1.00,1.12)	1.09 (0.94,1.27)			
Lag2	1.03***(1.02,1.04)	1.10***(1.07,1.13)	**1.15***(1.09,1.21)**	1.15 (0.98,1.33)			**1.12**
**(c)** ***All-cause mortality***							

Whole Taiwan							
Lag0	1.00***(1.00,1.01)	1.01** (1.00,1.02)	**1.03** (1.01,1.05)**				**1.03**
Lag1	1.00** (1.00,1.01)	1.03***(1.02,1.04)	**1.05***(1.03,1.08)**				**1.05**
Lag2	1.00 (1.00,1.00)	1.01 (1.00,1.02)	**1.03* (1.00,1.05)**				**1.03**
North Taiwan							
Lag0	1.00 (1.00,1.00)	1.01* (1.00,1.01)	1.01* (1.00,1.02)	1.01* (1.00,1.03)	1.03 (1.00,1.06)	1.13 (0.98,1.32)	
Lag1	1.00 (1.00,1.00)	1.01* (1.00,1.01)	1.01** (1.00,1.02)	1.02** (1.01,1.03)	**1.04** (1.01,1.07)**	1.03 (0.89,1.21)	**1.04**
Lag2	1.00 (1.00,1.00)	1.01* (1.00,1.01)	1.01 (1.00,1.01)	1.01 (1.00,1.02)	1.02 (0.99,1.06)	1.15 (0.98,1.33)	
Central Taiwan							
Lag0	1.01* (1.00,1.01)	1.00 (0.98,1.03)	1.01 (0.94,1.09)				
Lag1	1.00 (1.00,1.01)	1.01 (0.98,1.03)	1.01 (0.94,1.09)				
Lag2	1.00 (1.00,1.01)	1.01 (0.99,1.03)	1.01 (0.94,1.08)				
South Taiwan							
Lag0	1.00** (1.00,1.01)	1.04***(1.02,1.05)	**1.07***(1.03,1.12)**				**1.07**
Lag1	1.00* (1.00,1.01)	1.02* (1.00,1.04)	**1.05* (1.01,1.09)**				**1.05**
Lag2	1.00* (1.00,1.01)	1.02** (1.00,1.04)	**1.07** (1.02,1.11)**				**1.07**
East Taiwan							
Lag0	1.01* (1.00,1.02)	1.02 (0.99,1.05)	1.04 (0.98,1.10)	1.13 (0.95,1.35)			
Lag1	1.00 (0.99,1.01)	1.03 (1.00,1.07)	1.04 (0.98,1.11)	1.03 (0.86,1.23)			
Lag2	1.00 (0.99,1.01)	0.99 (0.96,1.02)	0.98 (0.93,1.04)	0.99 (0.84,1.18)			

$^{*}p < 0.05$ $^{**}p < 0.01$ $^{***}p < 0.001$; [a]CI: confidence interval. RaRR is reference-adjusted risk ratio, defined as RR (with statistical significance) of the threshold candidates against that of a reference (30°C); RaRR is shown only for the highest threshold candidate (in bold).

Table S3. Relative risks (RRs) of (a) heat-related emergency visits, (b) heat-related hospital visits, and (c) all-cause mortality at different temperature threshold candidates for different sex and age groups.

Threshold (°C)	RR (95%CI[a])						RaRR
	30	31	32	32.5	33	33.5	
		(a)	***Heat-related emergency visits***				
			Whole Taiwan				
Lag0	1.25***(1.24,1.26)	1.28***(1.26,1.29)	1.32***(1.29,1.34)	1.33***(1.30,1.36)	1.37***(1.33,1.42)	**1.66***(1.52,1.81)**	**1.33**
Lag1	1.06***(1.04,1.07)	1.07***(1.05,1.08)	1.08***(1.06,1.10)	1.09***(1.07,1.12)	1.11***(1.06,1.15)	**1.18***(1.07,1.29)**	**1.11**
Lag2	0.97***(0.96,0.98)	0.97***(0.96,0.98)	0.98** (0.96,0.99)	0.99 (0.96,1.01)	1.04* (1.00,1.08)	**1.11* (1.01,1.22)**	**1.14**
			Female				
Lag0	1.22***(1.19,1.24)	1.24***(1.21,1.27)	1.29***(1.24,1.34)	1.33***(1.27,1.39)	1.43***(1.33,1.54)	**1.95***(1.64,2.31)**	**1.60**
Lag1	1.04** (1.01,1.06)	1.06***(1.03,1.09)	1.09***(1.05,1.14)	1.11***(1.05,1.17)	1.15***(1.06,1.24)	**1.38***(1.15,1.65)**	**1.33**
Lag2	1.05***(1.03,1.07)	1.06***(1.04,1.09)	1.09***(1.05,1.13)	1.14***(1.08,1.19)	1.22***(1.13,1.31)	**1.31** (1.09,1.58)**	**1.25**
			Male				
Lag0	1.26***(1.25,1.28)	1.29***(1.27,1.31)	1.32***(1.30,1.35)	1.34***(1.30,1.37)	1.36***(1.30,1.42)	**1.59***(1.43,1.76)**	**1.26**
Lag1	1.06***(1.05,1.08)	1.07***(1.05,1.09)	1.07***(1.05,1.10)	1.09***(1.06,1.13)	1.09***(1.04,1.15)	1.11 (0.99,1.24)	
Lag2	0.95***(0.94,0.96)	0.95***(0.93,0.96)	0.94***(0.92,0.96)	0.95***(0.92,0.97)	0.99 (0.95,1.04)	1.06 (0.95,1.19)	
			Age 0-14				
Lag0	1.09***(1.05,1.12)	1.10***(1.05,1.15)	1.14***(1.07,1.23)	1.19***(1.08,1.31)	**1.26** (1.07,1.48)**	1.43 (0.94,2.18)	**1.16**
Lag1	1.05* (1.00,1.09)	1.06 (1.00,1.11)	1.08 (1.00,1.17)	1.10 (0.99,1.22)	1.14 (0.95,1.36)	1.45 (0.94,2.23)	
Lag2	1.00 (0.97,1.04)	1.02 (0.97,1.06)	1.04 (0.97,1.12)	1.08 (0.98,1.19)	**1.18* (1.00,1.39)**	1.34 (0.89,2.03)	**1.18**
			Age 15-64				
Lag0	1.27***(1.25,1.28)	1.29***(1.27,1.31)	1.33***(1.30,1.35)	1.35***(1.31,1.38)	1.39***(1.33,1.45)	**1.67***(1.51,1.84)**	**1.31**
Lag1	1.06***(1.05,1.08)	1.07***(1.05,1.09)	1.08***(1.05,1.10)	1.09***(1.06,1.12)	1.09***(1.04,1.15)	**1.16** (1.04,1.30)**	**1.09**
Lag2	0.96***(0.95,0.97)	0.96***(0.94,0.97)	0.96***(0.94,0.97)	0.97* (0.94,0.99)	1.01 (0.97,1.06)	1.07 (0.96,1.19)	
			Age ≥65				
Lag0	1.31***(1.27,1.34)	1.33***(1.29,1.38)	1.35***(1.29,1.41)	1.35***(1.27,1.43)	1.35***(1.24,1.48)	**1.67***(1.36,2.07)**	**1.27**
Lag1	1.03* (1.00,1.07)	1.05* (1.01,1.09)	1.07** (1.02,1.13)	1.12***(1.05,1.19)	**1.17** (1.06,1.29)**	1.17 (0.94,1.47)	**1.14**[a]
Lag2	1.02 (1.00,1.05)	1.04* (1.00,1.07)	1.06* (1.01,1.10)	1.07* (1.01,1.14)	1.15** (1.05,1.26)	**1.29* (1.04,1.61)**	**1.26**
		(b)	***Heat-related hospital visits***				
			Whole Taiwan				
Lag0	1.10***(1.10,1.10)	1.11***(1.10,1.11)	1.12***(1.12,1.12)	1.14***(1.13,1.14)	1.18***(1.17,1.19)	**1.31***(1.29,1.33)**	**1.19**
Lag1	1.03***(1.03,1.03)	1.04***(1.04,1.04)	1.06***(1.06,1.07)	1.08***(1.08,1.09)	1.10***(1.09,1.11)	**1.12***(1.10,1.14)**	**1.09**
Lag2	1.04***(1.04,1.04)	1.04***(1.04,1.04)	1.04***(1.04,1.04)	1.05***(1.04,1.05)	1.07***(1.06,1.08)	**1.15***(1.13,1.18)**	**1.11**
			Female				
Lag0	1.09***(1.09,1.09)	1.10***(1.10,1.10)	1.11***(1.11,1.12)	1.13***(1.13,1.14)	1.18***(1.17,1.19)	**1.31***(1.28,1.34)**	**1.20**
Lag1	1.02***(1.02,1.03)	1.04***(1.03,1.04)	1.06***(1.06,1.07)	1.08***(1.08,1.09)	1.10***(1.09,1.11)	**1.12***(1.09,1.15)**	**1.10**
Lag2	1.05***(1.04,1.05)	1.05***(1.05,1.05)	1.05***(1.04,1.05)	1.05***(1.05,1.06)	1.08***(1.07,1.09)	**1.16***(1.14,1.19)**	**1.10**
			Male				
Lag0	1.11***(1.10,1.11)	1.12***(1.11,1.12)	1.13***(1.12,1.13)	1.14***(1.13,1.15)	1.18***(1.17,1.19)	**1.32***(1.29,1.36)**	**1.19**
Lag1	1.03***(1.03,1.03)	1.04***(1.04,1.05)	1.07***(1.06,1.07)	1.09***(1.08,1.09)	1.10***(1.09,1.11)	**1.12***(1.09,1.15)**	**1.09**
Lag2	1.03***(1.03,1.04)	1.03***(1.03,1.04)	1.03***(1.03,1.04)	1.04***(1.03,1.05)	1.06***(1.05,1.07)	**1.14***(1.11,1.18)**	**1.11**

				Age 0-14			
Lag0	1.10***(1.09,1.11)	1.11***(1.10,1.12)	1.12***(1.10,1.14)	1.14***(1.12,1.17)	1.19***(1.15,1.24)	**1.25***(1.14,1.37)**	**1.14**
Lag1	1.03***(1.01,1.04)	1.04***(1.02,1.05)	1.07***(1.05,1.10)	1.09***(1.06,1.12)	1.11***(1.06,1.15)	**1.19***(1.08,1.31)**	**1.16**
Lag2	1.04***(1.03,1.05)	1.05***(1.03,1.06)	1.04***(1.03,1.06)	1.06***(1.04,1.09)	1.10***(1.06,1.14)	**1.21***(1.10,1.33)**	**1.16**
				Age 15-64			
Lag0	1.10***(1.10,1.10)	1.11***(1.11,1.11)	1.12***(1.12,1.13)	1.14***(1.14,1.15)	1.19***(1.18,1.19)	**1.32***(1.30,1.35)**	**1.20**
Lag1	1.03***(1.03,1.03)	1.04***(1.04,1.04)	1.06***(1.06,1.07)	1.08***(1.08,1.09)	1.10***(1.09,1.11)	**1.11***(1.09,1.14)**	**1.08**
Lag2	1.04***(1.04,1.04)	1.04***(1.04,1.04)	1.04***(1.04,1.04)	1.04***(1.04,1.05)	1.07***(1.06,1.08)	**1.15***(1.13,1.18)**	**1.11**
				Age ≥65			
Lag0	1.08***(1.07,1.09)	1.08***(1.07,1.09)	1.09***(1.07,1.10)	1.09***(1.08,1.11)	1.12***(1.10,1.14)	**1.24***(1.18,1.31)**	**1.15**
Lag1	1.02***(1.01,1.03)	1.03***(1.02,1.04)	1.06***(1.05,1.07)	1.09***(1.07,1.10)	1.11***(1.09,1.14)	**1.15***(1.09,1.22)**	**1.13**
Lag2	1.04***(1.04,1.05)	1.04***(1.04,1.05)	1.05***(1.03,1.06)	1.05***(1.04,1.07)	1.07***(1.05,1.09)	**1.15***(1.09,1.21)**	**1.11**
			(c) ***All-cause mortality***				
				Whole Taiwan			
Lag0	1.00***(1.00,1.01)	1.00***(1.00,1.01)	1.01***(1.00,1.01)	1.01***(1.01,1.02)	1.02***(1.01,1.03)	**1.05***(1.02,1.07)**	**1.05**
Lag1	1.00 (1.00,1.00)	1.00* (1.00,1.01)	1.01** (1.00,1.01)	1.01* (1.00,1.02)	1.01* (1.00,1.02)	**1.04** (1.01,1.07)**	**1.04**
Lag2	1.00* (1.00,1.00)	1.00* (1.00,1.01)	1.01***(1.00,1.01)	1.01***(1.01,1.02)	1.02***(1.01,1.03)	**1.04** (1.02,1.07)**	**1.04**
				Female			
Lag0	1.00 (1.00,1.01)	1.00 (1.00,1.01)	1.00 (1.00,1.01)	1.01** (1.00,1.02)	1.03***(1.01,1.04)	**1.04* (1.00,1.09)**	**1.04**
Lag1	1.00 (1.00,1.01)	1.01* (1.00,1.01)	1.01** (1.00,1.02)	1.01** (1.00,1.02)	1.01 (1.00,1.03)	1.04 (1.00,1.09)	
Lag2	1.00* (1.00,1.01)	1.01* (1.00,1.01)	1.01* (1.00,1.01)	1.01* (1.00,1.02)	1.02* (1.00,1.04)	**1.04* (1.00,1.09)**	**1.04**
				Male			
Lag0	1.01***(1.00,1.01)	1.01***(1.00,1.01)	1.01***(1.00,1.02)	1.01***(1.01,1.02)	1.02** (1.01,1.03)	**1.05** (1.01,1.09)**	**1.04**
Lag1	1.00 (1.00,1.00)	1.00 (1.00,1.01)	1.00 (1.00,1.01)	1.00 (1.00,1.01)	1.01 (1.00,1.03)	1.04 (1.00,1.07)	
Lag2	1.00 (1.00,1.00)	1.00 (1.00,1.01)	1.01** (1.00,1.01)	1.01***(1.01,1.02)	1.02** (1.01,1.03)	**1.05** (1.01,1.08)**	**1.05**
				Age 0-14			
Lag0	1.01 (0.98,1.03)	1.00 (0.97,1.03)	0.98 (0.93,1.02)	0.97 (0.90,1.04)	0.95 (0.84,1.07)	0.91 (0.65,1.27)	
Lag1	0.99 (0.96,1.02)	1.00 (0.96,1.04)	1.03 (0.97,1.09)	1.03 (0.95,1.12)	1.06 (0.93,1.21)	1.22 (0.87,1.71)	
Lag2	1.00 (0.98,1.02)	1.00 (0.97,1.03)	0.99 (0.94,1.04)	0.99 (0.92,1.06)	0.95 (0.84,1.08)	0.82 (0.58,1.15)	
				Age 15-64			
Lag0	1.00* (1.00,1.01)	1.01 (1.00,1.01)	1.01 (1.00,1.02)	1.01* (1.00,1.03)	1.03** (1.01,1.05)	**1.08** (1.03,1.14)**	**1.08**
Lag1	1.00 (0.99,1.00)	1.00 (0.99,1.00)	1.00 (0.99,1.01)	1.00 (0.98,1.01)	1.00 (0.98,1.02)	1.01 (0.96,1.07)	
Lag2	1.00* (1.00,1.01)	1.01* (1.00,1.01)	1.01* (1.00,1.02)	1.02** (1.00,1.03)	1.02* (1.00,1.04)	1.05 (0.99,1.10)	
				Age ≥65			
Lag0	1.00***(1.00,1.01)	1.01** (1.00,1.01)	1.01** (1.00,1.01)	1.01***(1.01,1.02)	1.02***(1.01,1.03)	**1.04* (1.01,1.07)**	**1.04**
Lag1	1.00** (1.00,1.01)	1.01***(1.00,1.01)	1.01***(1.00,1.02)	1.01** (1.00,1.02)	1.02** (1.01,1.03)	**1.05** (1.01,1.08)**	**1.05**
Lag2	1.00 (1.00,1.00)	1.00 (1.00,1.01)	1.01** (1.00,1.01)	1.01** (1.00,1.02)	1.02***(1.01,1.03)	**1.05** (1.01,1.08)**	**1.05**

$^{*}p < 0.05$ $^{**}p < 0.01$ $^{***}p < 0.001$, [a]CI: confidence interval, RaRR is reference-adjusted risk ratio, defined as RR (with statistical significance) of the threshold candidates against that of a reference (30°C); RaRR is shown only for the highest threshold candidate (in bold).

Table S4. Relative risks (RRs) of (a) heat-related emergency visits, (b) heat-related hospital visits, and (c) all-cause mortality at different temperature threshold candidates for different sub-regions.

Threshold (°C)	RR (95%CI[a])								RaRR
	30	32	32.5	33	33.5	34	34.5	35	
			(a) ***Heat-related emergency visits***						
				Whole Taiwan					
Lag0	1.25***(1.24,1.26)	1.32***(1.29,1.34)	1.33***(1.30,1.36)	1.37***(1.33,1.42)	**1.66***(1.52,1.81)**				**1.33**
Lag1	1.06***(1.04,1.07)	1.08***(1.06,1.10)	1.09***(1.07,1.12)	1.11***(1.06,1.15)	**1.18***(1.07,1.29)**				**1.11**
Lag2	0.97***(0.96,0.98)	0.98** (0.96,0.99)	0.99 (0.96,1.01)	1.04* (1.00,1.08)	**1.11* (1.01,1.22)**				**1.14**
				North Taiwan					
Lag0	1.17***(1.16,1.19)	1.19***(1.17,1.20)	1.19***(1.17,1.21)	1.20***(1.18,1.22)	1.22***(1.19,1.25)	1.26***(1.22,1.31)	1.41***(1.33,1.49)	**2.08***(1.77,2.43)**	**1.78**
Lag1	1.05***(1.04,1.06)	1.05***(1.04,1.07)	1.06***(1.04,1.07)	1.05***(1.03,1.08)	1.06***(1.03,1.09)	1.08***(1.04,1.12)	1.12***(1.05,1.20)	**1.39***(1.16,1.65)**	**1.32**
Lag2	1.00 (0.99,1.01)	1.01 (1.00,1.02)	1.01 (0.99,1.03)	1.01 (0.99,1.03)	1.02 (0.99,1.04)	1.03 (0.99,1.07)	1.09* (1.02,1.17)	**1.38***(1.15,1.65)**	**1.38**
				Central Taiwan					
Lag0	1.21***(1.19,1.23)	1.23***(1.20,1.27)	1.24***(1.20,1.29)	1.31***(1.24,1.39)	1.52***(1.35,1.71)	**2.39***(1.62,3.51)**			**1.98**
Lag1	1.06***(1.04,1.07)	1.07***(1.04,1.10)	1.09***(1.04,1.13)	1.13***(1.06,1.21)	**1.28***(1.12,1.46)**	1.10 (0.66,1.84)			**1.21**

Lag2	1.03** (1.01,1.04)	1.07***(1.04,1.10)	1.10***(1.06,1.15)	1.17***(1.09,1.24)	1.32***(1.15,1.51)	**2.00** (1.24,3.24)**			**1.94**
				South Taiwan					
Lag0	1.30***(1.28,1.33)	1.35***(1.31,1.39)	1.39***(1.34,1.44)	1.42***(1.35,1.49)	1.55***(1.42,1.69)	**1.88***(1.46,2.43)**			**1.45**
Lag1	1.07***(1.04,1.09)	1.09***(1.06,1.13)	1.08***(1.04,1.13)	1.10** (1.04,1.17)	1.10 (0.99,1.21)	1.30 (0.99,1.72)			
Lag2	0.92***(0.90,0.93)	0.90***(0.87,0.93)	0.91***(0.87,0.95)	0.92** (0.87,0.98)	0.97 (0.88,1.07)	1.06 (0.80,1.41)			
				East Taiwan					
Lag0	1.27***(1.23,1.31)	1.27***(1.20,1.35)	1.25***(1.16,1.36)	1.19* (1.03,1.38)	1.06 (0.68,1.65)				
Lag1	1.02 (0.99,1.06)	1.02 (0.96,1.09)	1.04 (0.95,1.14)	1.14 (0.98,1.33)	1.41 (0.94,2.09)				
Lag2	0.96* (0.93,0.99)	0.97 (0.91,1.03)	0.99 (0.91,1.08)	1.03 (0.88,1.20)	1.35 (0.90,2.03)				
				(b) Heat-related hospital visits					
				Whole Taiwan					
Lag0	1.10***(1.10,1.10)	1.12***(1.12,1.12)	1.14***(1.13,1.14)	1.18***(1.17,1.19)	**1.31***(1.29,1.33)**				**1.19**
Lag1	1.03***(1.03,1.03)	1.06***(1.06,1.07)	1.08***(1.08,1.09)	1.10***(1.09,1.11)	**1.12***(1.10,1.14)**				**1.09**
Lag2	1.04***(1.04,1.04)	1.04***(1.04,1.04)	1.05***(1.04,1.05)	1.07***(1.06,1.08)	**1.15***(1.13,1.18)**				**1.11**
				North Taiwan					
Lag0	1.07***(1.07,1.07)	1.08***(1.07,1.08)	1.08***(1.08,1.08)	1.08***(1.08,1.09)	1.09***(1.09,1.10)	1.11***(1.10,1.12)	1.16***(1.14,1.17)	**1.29***(1.25,1.32)**	**1.21**
Lag1	1.03***(1.03,1.03)	1.04***(1.04,1.04)	1.04***(1.04,1.04)	1.04***(1.04,1.05)	1.05***(1.05,1.06)	1.07***(1.06,1.07)	1.11***(1.09,1.12)	**1.33***(1.29,1.37)**	**1.29**
Lag2	1.04***(1.03,1.04)	1.04***(1.04,1.04)	1.04***(1.04,1.04)	1.04***(1.04,1.04)	1.04***(1.04,1.05)	1.05***(1.05,1.06)	1.09***(1.08,1.10)	**1.27***(1.24,1.31)**	**1.22**
				Central Taiwan					
Lag0	1.08***(1.08,1.08)	1.09***(1.08,1.09)	1.09***(1.09,1.10)	1.11***(1.10,1.12)	**1.13***(1.11,1.16)**	1.13***(1.05,1.22)			**1.05**
Lag1	1.04***(1.03,1.04)	1.05***(1.05,1.06)	1.06***(1.05,1.07)	1.08***(1.07,1.09)	**1.09***(1.07,1.11)**	0.90** (0.83,0.97)			**1.05**
Lag2	1.05***(1.04,1.05)	1.06***(1.06,1.07)	1.07***(1.07,1.08)	1.10***(1.09,1.12)	**1.15***(1.13,1.17)**	1.12** (1.04,1.20)			**1.10**
				South Taiwan					
Lag0	1.11***(1.10,1.12)	1.14***(1.13,1.16)	1.16***(1.15,1.18)	1.19***(1.16,1.21)	1.24***(1.20,1.28)	**1.47***(1.34,1.61)**			**1.32**
Lag1	1.04***(1.03,1.04)	1.08***(1.06,1.09)	1.09***(1.07,1.10)	1.11***(1.09,1.13)	1.15***(1.11,1.19)	**1.52***(1.37,1.68)**			**1.45**
Lag2	1.02***(1.01,1.02)	1.02***(1.01,1.03)	1.03***(1.02,1.05)	1.05***(1.03,1.07)	1.11***(1.07,1.14)	**1.35***(1.22,1.49)**			**1.32**
				East Taiwan					
Lag0	1.14***(1.13,1.15)	1.15***(1.12,1.17)	1.13***(1.10,1.16)	1.10***(1.04,1.16)	**1.17* (1.01,1.35)**				**1.03**
Lag1	1.04***(1.03,1.05)	1.05***(1.02,1.07)	1.05** (1.02,1.09)	1.10***(1.04,1.17)	**1.52***(1.31,1.76)**				**1.46**
Lag2	1.02***(1.01,1.03)	1.04***(1.02,1.06)	1.07***(1.04,1.11)	1.15***(1.09,1.21)	**1.43***(1.26,1.64)**				**1.40**
				(c) All-cause mortality					
				Whole Taiwan					
Lag0	1.00***(1.00,1.01)	1.01***(1.00,1.01)	1.01***(1.01,1.02)	1.02***(1.01,1.03)	**1.05***(1.02,1.07)**				**1.05**
Lag1	1.00 (1.00,1.00)	1.01** (1.00,1.01)	1.01* (1.00,1.02)	1.01* (1.00,1.02)	**1.04** (1.01,1.07)**				**1.04**
Lag2	1.00* (1.00,1.00)	1.01***(1.00,1.01)	1.01***(1.01,1.02)	1.02***(1.01,1.03)	**1.04** (1.02,1.07)**				**1.04**
				North Taiwan					
Lag0	1.00* (1.00,1.00)	1.00** (1.00,1.01)	1.01***(1.00,1.01)	1.01***(1.00,1.01)	1.01***(1.01,1.02)	1.02** (1.01,1.02)	1.03** (1.01,1.04)	**1.07** (1.02,1.13)**	**1.07**
Lag1	1.00 (1.00,1.00)	1.00 (1.00,1.00)	1.00 (1.00,1.00)	1.00 (1.00,1.01)	1.00 (1.00,1.01)	1.01 (1.00,1.02)	1.01 (0.99,1.03)	1.02 (0.97,1.08)	

Lag2	1.00* (1.00,1.00)	1.01***(1.00,1.01)	1.01***(1.00,1.01)	1.01***(1.00,1.01)	1.01***(1.00,1.02)	1.01* (1.00,1.02)	1.02* (1.00,1.04)	**1.07* (1.01,1.12)**	**1.07**
				Central Taiwan					
Lag0	1.00 (1.00,1.01)	1.01 (1.00,1.01)	1.01 (1.00,1.02)	1.02* (1.00,1.04)	1.04 (0.99,1.08)	1.12 (0.96,1.31)			
Lag1	1.01* (1.00,1.01)	1.01* (1.00,1.02)	1.01 (0.99,1.02)	1.00 (0.98,1.01)	1.00 (0.96,1.04)	1.00 (0.85,1.18)			
Lag2	1.00 (1.00,1.00)	1.00 (0.99,1.01)	1.00 (0.99,1.01)	1.01 (0.99,1.03)	1.03 (0.99,1.07)	1.15 (0.98,1.35)			
				South Taiwan					
Lag0	1.00 (1.00,1.01)	1.01** (1.00,1.01)	1.01** (1.00,1.02)	1.02***(1.01,1.03)	1.04***(1.02,1.06)	**1.10** (1.03,1.18)**			**1.10**
Lag1	1.00* (1.00,1.01)	1.01* (1.00,1.01)	1.01 (1.00,1.02)	1.01 (1.00,1.02)	1.01 (0.98,1.03)	1.00 (0.94,1.08)			
Lag2	1.00 (1.00,1.00)	1.01 (1.00,1.01)	1.01* (1.00,1.02)	1.01 (1.00,1.02)	1.01 (0.99,1.04)	1.06 (0.99,1.13)			
				East Taiwan					
Lag0	1.01* (1.00,1.02)	1.02 (1.00,1.04)	1.02 (0.99,1.05)	1.03 (0.97,1.09)	1.11 (0.94,1.31)				
Lag1	1.00 (0.99,1.01)	1.01 (0.99,1.04)	1.03 (1.00,1.07)	1.08* (1.02,1.15)	**1.20* (1.02,1.42)**				**1.20**
Lag2	1.00 (0.99,1.01)	1.00 (0.98,1.02)	1.00 (0.97,1.03)	0.99 (0.93,1.05)	1.04 (0.88,1.23)				

*$p < 0.05$** $p < 0.01$*** $p < 0.001$, [a]CI: confidence interval, RaRR is reference-adjusted risk ratio, defined as RR (with statistical significance) of the threshold candidates against that of a reference (30°C); RaRR is shown only for the highest threshold candidate (in bold).

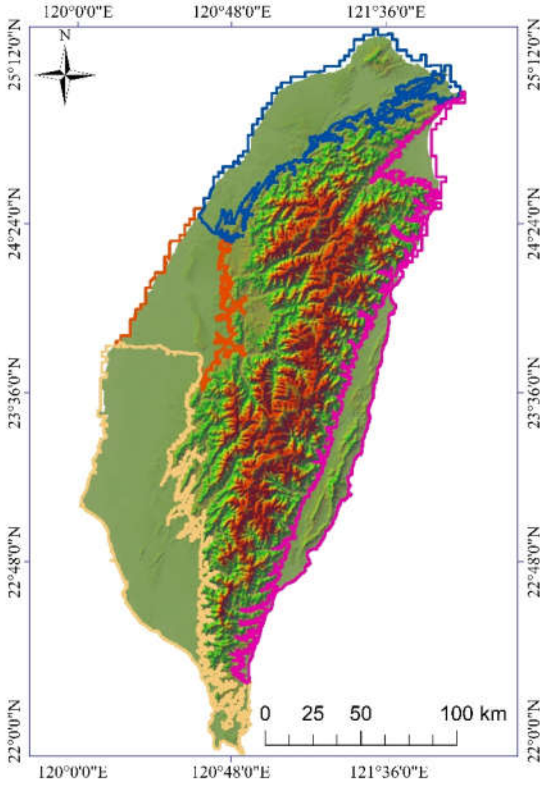

Figure S1. Taiwan island divided into sub-regions of North, Central, South, and East Taiwan.
